# Disseminated *Mycobacterium chimaera* infection favoring the development of Kaposi’s sarcoma: a case report

**DOI:** 10.1186/s12941-022-00547-x

**Published:** 2022-12-09

**Authors:** Tommaso Clemente, Vincenzo Spagnuolo, Martina Bottanelli, Marco Ripa, Benedetto Del Forno, Elena Busnardo, Giuseppe Di Lucca, Antonella Castagna, Anna Danise

**Affiliations:** 1 School of Medicine and Surgery, Vita-Salute San Raffaele University, Via Stamira D’Ancona, 20, 20127 Milan, Italy; 2 Infectious Diseases, IRCCS San Raffaele Scientific Institute, Milan, Italy; 3 Department of Cardiac Surgery, IRCCS San Raffaele Scientific Institute, Milan, Italy; 4 Myocarditis Disease Unit, IRCCS San Raffaele Scientific Institute, Milan, Italy; 5 Department of Nuclear Medicine, IRCCS San Raffaele Scientific Institute, Milan, Italy; 6 General Medicine and Advanced Care Unit, IRCCS San Raffaele Scientific Institute, Milan, Italy

**Keywords:** Mycobacterium chimaera, Non-tuberculous mycobacteria, Infective endocarditis, HHV8, Kaposi

## Abstract

**Background:**

Disseminated *Mycobacterium chimaera* infection is an emerging disease in people undergone to cardiothoracic surgery, which need to be suspected also with atypical presentations.

**Case presentation:**

We report the case of a 74-year-old man with fever of unknown origin, purple nodules on both feet and a history of open-heart surgery. Imaging investigations showed an abscess near aortic bioprosthesis but screening for endocarditis resulted negative and pyrexia did not respond to antibiotic therapy. A biopsy of cutaneous lesions showed HHV8-related Kaposi’s sarcoma, so bone marrow biopsy was executed with evidence of HHV8 localization. Bone marrow and urine mycobacterial cultures resulted positive for *M. chimaera* and a specific antimicrobial therapy was started, with apyrexia after 7 weeks.

**Conclusions:**

*M. chimaera* infection should be always investigated as a possible etiology of fever of unknow origin in people with a history of open-heart surgical intervention, even with negative mycobacterial blood cultures. The possible role of disseminated infection in inducing immunodepression with the occurrence of other opportunistic diseases (such as Kaposi’s sarcoma) cannot be excluded.

## Background

*Mycobacterium chimaera* is an opportunistic, water-borne, non-tuberculous mycobacterium (NTM), belonging to the *Mycobacterium avium* complex, first described in 2004 [1]. It has been associated with pulmonary infections, mainly in immunocompromised patients or in individuals with underlying respiratory diseases [2], but it seems to be less virulent than other NTM [3].

In 2013 the first cases of *M. chimaera* infections in patients with a history of cardiothoracic surgery, especially open-heart surgery, were reported [4]. After a latent period of months to years, this pathogen may cause prosthetic valve endocarditis (PVE), surgical site infection, vascular graft infection or disseminated disease, with a high case fatality rate [4–7]. It is primarily acquired via contaminated bioaerosols emitted from heater-cooler units, thermoregulatory components of extracorporeal membrane oxygenation (ECMO) systems [6, 8]. The spreading of ECMO systems has made post-surgical *M. chimaera* infections a global health problem: all over the world, more than 140 cases of severe infection following cardiothoracic surgery have been described until 2019 [9]. The diagnosis is often difficult, due to non-specific signs, symptoms and laboratory features, including fever, dyspnea, fatigue, weight loss, pancytopenia, and elevation of C-reactive protein (CRP), transaminases and creatinine [5]. The diagnostic process may be further slowed down in the case of a concomitant other disease.

To our knowledge, this is the first reported case of disseminated *M. chimaera* infection with concurrent Kaposi’s sarcoma (KS) in a patient who underwent open-heart surgery. In this work, we outline the diagnostic process and try to suggest an explanation to the coexistence of the two conditions.

## Case presentation

**Fig. 1:**
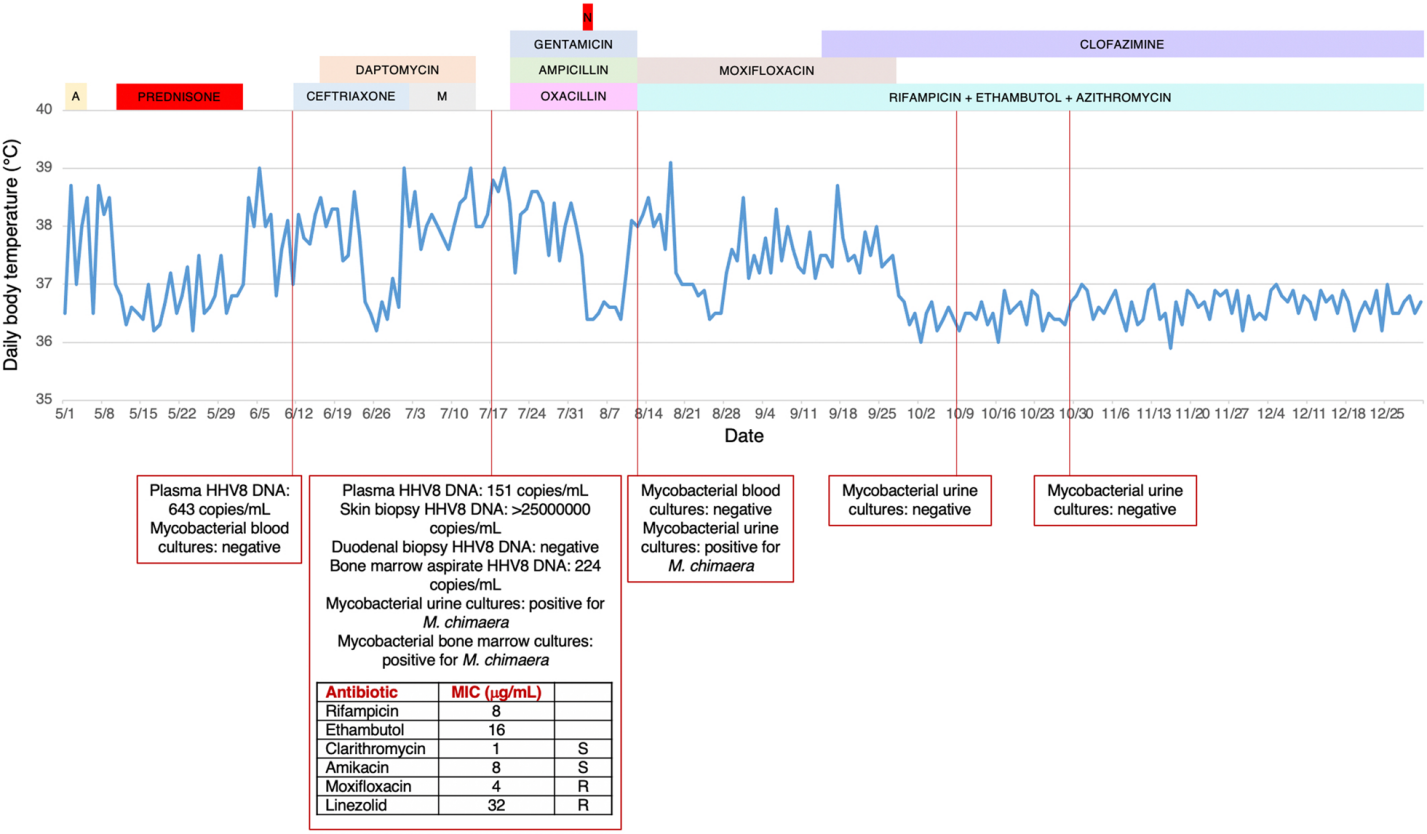
Patient’s daily body temperature, treatments and microbiological investigations. The highest daily body temperature from May to December 2021 was reported in degrees Celsius (°C). Only concurrent antibiotic and anti-inflammatory treatments and microbiological investigations inherent to mycobacterial and human herpesvirus 8 (HHV8) infections were shown. In the susceptibility test, susceptibility and resistance to a specific antibiotic were evaluated comparing minimum inhibitory concentration to Clinical and Laboratory Standard Institute clinical breakpoints, when available [10]. *A* amoxicillin-clavulanic acid, *HHV8* human herpesvirus 8, *M* meropenem, *MIC* minimum inhibitory concentration, *N* naproxen, *R* resistant, *S* susceptible

**Fig. 2:**
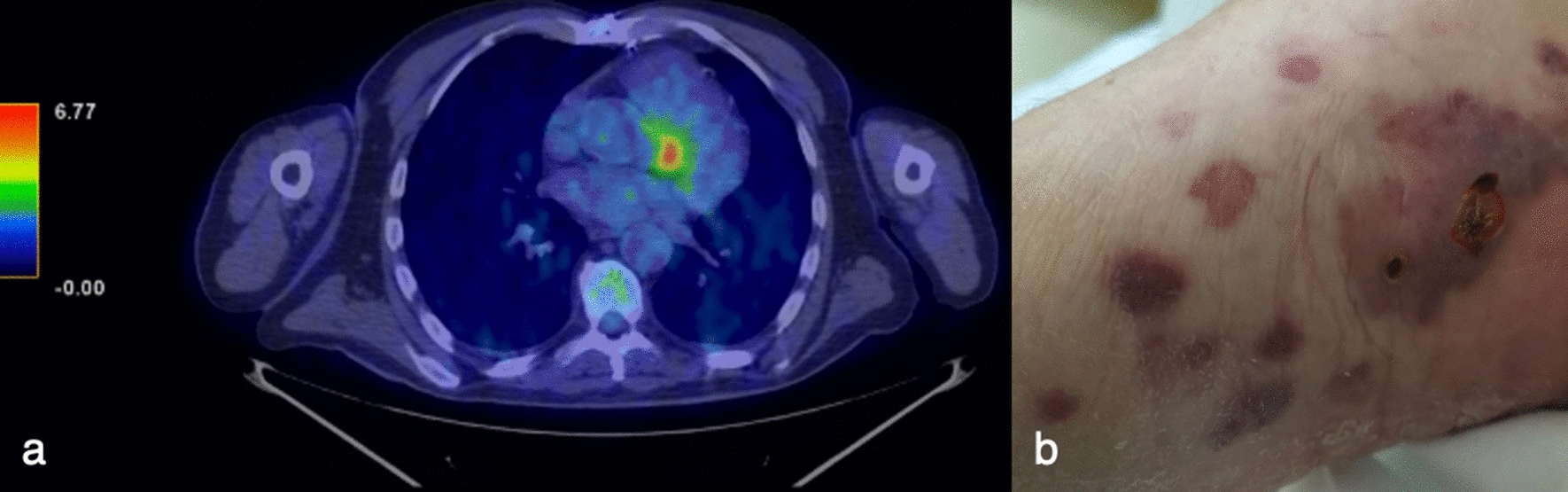
Heart and skin findings.** a** Total body Fluorine-18-fluorodeoxyglucose positron emission tomography executed in June 2021 showing an accumulation of the tracer near the aortic bioprosthesis. **b** Cutaneous lesions on the right foot of the patient, which were biopsied in July 2021

In 2017 in the Department of Cardiac Surgery, IRCCS San Raffaele Scientific Institute (Milan, Italy), a 70-year-old man underwent coronary artery bypass graft and aortic valve replacement with bioprosthesis, needing extracorporeal circulation. His past medical history included hypertension and chronic kidney disease. In June 2021 he was hospitalized in our Infectious Diseases Unit for pyrexia from more than one month (Fig. 1), weight loss and suspected PVE. Transthoracic echocardiography (TTE) had shown mobile vegetations on aortic bioprosthesis, that had not been confirmed at transesophageal echocardiography (TEE). During the hospitalization, TTE and TEE were repeated and did not confirm the diagnosis of PVE. However, total body Fluorine-18-fluorodeoxyglucose positron emission tomography (18F-FDG PET) detected an accumulation of the tracer near the aortic bioprosthesis (Fig. 2a). Coronary computed tomography angiography and gadolinium-enhanced cardiac magnetic resonance revealed a periprosthetic aortic abscess. Complete blood count showed anemia, thrombocytopenia, leucopenia and lymphopenia (with a lymphocyte nadir of 400 cells/µL, CD4^+^ T-cell count of 195 cells/µL, CD8^+^ T-cell count of 202 cells/µL and CD4^+^/CD8^+^ ratio of 0.97), and blood chemistry tests revealed an elevation in CRP and interleukin (IL)-6 concentrations (with peaks of 65.2 mg/L and 42.2 pg/mL, respectively), hypoalbuminemia and a reduction in estimated glomerular filtration rate. Blood culture series and screening for blood-culture-negative infective endocarditis [including liquid (BD BACTEC Mycobacteria Growth Indicator Tube 960 system; 2 sets) and solid (Lowenstein-Jensen; 2 sets) medium-based mycobacterial blood culture, Wright and Widal seroagglutination reaction, serology for *Brucella*, *Coxiella burnetii*, *Bartonella henselae*, *Mycoplasma pneumoniae*, and *Legionella pneumophila*, urine-based antigen detection for *Streptococcus pneumoniae* and *L. pneumophila*, plasma-based antigen detection for *Aspergillus* and *Cryptococcus*, histology for Whipple disease and real-time polymerase chain reaction (rtPCR) for *Tropheryma whipplei* on multiple duodenal biopsies] were negative. We also performed screening for non-infective endocarditis (including tests for antinuclear, anticardiolipin, and anti-β_2_-glycoprotein1 antibodies and lupus anticoagulant), with a negative result. Because of the persistence of fever despite multiple antibiotic treatments (Fig. 1), other causes of pyrexia were considered: screening for viral infections (including severe acute respiratory syndrome coronavirus 2 nasopharyngeal swab) was characterized only by a low-titer positivity of rtPCR for human herpesvirus 8 (HHV8) on plasma samples. The patient had no lymphadenopathies, pleural, pericardial or peritoneal effusions and hepatomegaly, but only moderate splenomegaly and some purple cutaneous nodular lesions localized to both feet (Fig. 2b), observed for the first time at the third day of hospitalization. Therefore, these nodules were biopsied and both histological analysis and rtPCR for HHV8 were consistent with KS (Fig. 1). Fourth generation HIV antigen/antibody combination assay was negative, letting us diagnose Classic KS. Histology and rtPCR for *B. henselae* on skin biopsies excluded also a possible case of bacillary angiomatosis. No lesions consistent with KS were revealed from esophagogastroduodenoscopy and HHV8 was not detected from duodenal biopsies (Fig. 1). Even though HHV8 viremia was < 1000 copies/mL (Fig. 1), clinical presentation and hemato-chemistry tests were consistent with KS inflammatory cytokine syndrome (KICS)-like manifestation. We performed bone marrow biopsy, which revealed a reduced T- and B-lymphocyte count and confirmed involvement by HHV8, and a naproxen test, with apyrexia for 5 days (Fig. 1). Moreover, although in our Institute no cases of *M. chimaera* endocarditis following cardiothoracic surgery were reported, in light of the clinical presentation and the previous exposition to heater-cooler systems, mycobacterial urine (BD BACTEC Mycobacteria Growth Indicator Tube 960 system and Lowenstein-Jensen medium), stool (BD BACTEC Mycobacteria Growth Indicator Tube 960 system and Lowenstein-Jensen medium) and bone marrow (BacT/ALERT^®^3D automated culture system) cultures were executed, with isolation of *M. chimaera* from urine and bone marrow (Fig. 1). According to European [11] and international [12] diagnostic criteria, we diagnosed a disseminated *M. chimaera* infection and oral antimycobacterial therapy was started, composed of a cornerstone with azithromycin 500 mg/day, ethambutol 1200 mg/day, and rifampicin 600 mg/day, plus moxifloxacin 400 mg/day, considering the multiorgan involvement (Fig. 1). At day 7 from treatment initiation, the therapy was upgraded adding clofazimine 100 mg/day for persistence of fever (Fig. 1). A multidisciplinary evaluation excluded indications to a cardiac surgical intervention, due to the absence of hemodynamic instability and prosthetic dysfunction, and given the high surgical risk. At day 34, with the result of susceptibility test, that showed *M. chimaera* susceptible to amikacin and clarithromycin, resistant to linezolid and moxifloxacin and with a minimum inhibitory concentration of 4 µg/mL for rifampicin and 16 µg/mL for ethambutol in absence of Clinical and Laboratory Standards Institute clinical breakpoints, moxifloxacin was stopped (Fig. 1). From week 7, the patient became apyretic (Fig. 1), blood tests remained stable except for a further reduction in estimated glomerular filtration rate (with the need to reduce ethambutol dosage to 1600 mg/2 days), and the cutaneous lesions did not expand. Oncologic evaluations at week 5, 26 and 40 excluded current indication to chemotherapy or other treatment for KS. At week 9 mycobacterial urine cultures became negative (Fig. 1).

## Discussion and conclusions

Since 2013, *M. chimaera* infections in patients subjected to cardiothoracic surgery with the use of heater-cooler units have become a global public health problem. These infections may be localized in surgical site or disseminated, with manifestations that can include splenomegaly, bone marrow and/or pulmonary involvement, choroidoretinitis, osteomyelitis, arthritis, hepatitis, nephritis, and myocarditis [5]. Despite the presence of specific diagnostic criteria [11, 12], difficulties in diagnosis seem to be due to the long period of latency between the surgical intervention and the first signs of infection [6], the non-specific clinical and laboratory features [5], the negativity of the classic microbiological investigations unable to detect mycobacteria [4–7], and the possible absence of pathologic findings at echocardiography [13]. A delay in the diagnostic process, together with the high risk of reintervention, the intrinsic antimicrobic resistance of these mycobacteria, and the low antibiotic penetration of the classic sites of infection, may make *M. chimaera* life-threatening [12].

In this work, we report the first case of *M. chimaera* infection after a surgical intervention performed in our Institute. The suspicious of a mycobacterial disease came from the aspecific clinical and laboratory manifestations, the plausible periprosthetic aortic abscess and the timing of heart surgery. In fact, in Italy the first case was described in 2016 and involved a woman with a disseminated infection and vertebral osteomyelitis following open-heart surgery [14]. However, in our case mycobacterial blood cultures (2 sets in both liquid and solid medium) resulted negative. Diagnostic sensitivity for mycobacterial blood cultures had been described as relatively low [15], so we searched for the pathogen in other samples and isolated it from bone marrow and urine, documenting a disseminated infection. Therefore, our case suggests that *M. chimaera* infection should be suspected in all patients with previous exposure to heater-cooler units, even when no other cases have been described in the same Institute and mycobacterial blood cultures result negative.

Furthermore, in the reported case, diagnosis became more difficult due to the overlap of mycobacterial infection and Mediterranean KS. Actually, first-line microbiological investigations for endocarditis were negative, cardiac imaging was not univocal for a periprosthetic abscess, and KICS-like presentation needed to be considered as a possible differential diagnosis, in light of initial clinical and laboratory findings. Severe inflammatory symptoms without the evidence of multicentric Castleman disease (MCD) were first described in an Italian immunocompetent HIV-negative woman with HHV8 infection, who developed KS after 10 months from the appearance of fever [16]. After 5 years a similar presentation was reported in 6 people with HIV/HHV8 coinfection, whose levels of HHV8 viremia, virally encoding IL-6 homolog (vIL-6), human IL-6 (hIL-6), and IL-10 were comparable to those seen in individuals with HIV and MCD, resulting significantly higher compared to subjects with HIV and only KS [17]. Therefore, it was suggested that the pathophysiology of KICS might be explained as a result of vIL-6 production and HHV8-mediated induction of host inflammatory cytokines, especially during the lytic phase of the infection. Consequently, a working case definition, based on symptoms, laboratory and radiographic abnormalities, evidence of systemic inflammation and of HHV8 viral activity, and exclusion of MCD was defined [18]. Even though our patient presented with KS, fever, mild splenomegaly, pancytopenia, elevation of CRP, and no evidence of MCD, he did not fulfil criteria for KICS, because of plasma HHV8 viral load, that was < 1000 copies/mL. Negativity of HIV test made further unlikely the diagnosis of KICS-like presentation. Administration of naproxen for 3 days (naproxen test) was followed by apyrexia, suggesting that our patient’s fever was partially justified by KS-associated manifestations, but a rtPCR for HHV8 on bone marrow plasma sample confirmed only a low titer positivity. Therefore, we could not definitely diagnose KICS-like manifestation.

As what concerns the pathogenesis of KS in our patient, Classic Mediterranean KS occurs typically in middle-aged and elderly HIV-negative people with HHV8 infection and is characterized by purple papules or nodules predominating in the lower limbs [19]. In this case, whether disseminated *M. chimaera* infection contributed to the occurrence of KS is unknown. Actually, mycobacterial bone marrow involvement might have had a role in the development of pancytopenia and reduced bone marrow and peripheral blood lymphocyte count with inverted CD4^+^/CD8^+^ ratio, which in turn could have favored the appearance of HHV8-related manifestations, similarly to what had been described in people living with HIV and in individuals with organ or bone marrow transplantation [20].

In conclusion, the case reported underlines the necessity of a complex diagnostic workflow to exclude *M. chimaera* infection in people with fever of unknown origin and previous cardiothoracic surgical intervention with need of extracorporeal circulation, even when initial mycobacterial blood cultures and echocardiographic investigations are negative. The absence of other cases reported in the same Institute cannot let the clinicians reject this diagnosis. Disseminated mycobacterial infection with bone marrow involvement might also explain, partially at least, a condition of immunodepression and therefore coexist with opportunistic diseases, as KS.

## Data Availability

Not applicable.

## Abbreviations

CRP: C-reactive protein
ECMO: Extracorporeal membrane oxygenation
hIL-6: Human interleukin 6
HHV8: Human herpesvirus 8
IL: Interleukin
KICS: Kaposi’s sarcoma inflammatory cytokine syndrome
KS: Kaposi’s sarcoma
MCD: Multicentric Castleman disease
NTM: Non-tuberculous mycobacterium
rtPCR: Real-time polymerase chain reaction
PVE: Prosthetic valve endocarditis
TEE: Transesophageal echocardiography
TTE: Transthoracic echocardiography
vIL-6: Virally encoding IL-6 homolog
18F-FDG PET: Total body fluorine-18-fluorodeoxyglucose positron emission tomography
